# The Comprehensive Analysis of N6-Methyadenosine Writer METTL3 and METTL14 in Gastric Cancer

**DOI:** 10.1155/2023/9822995

**Published:** 2023-02-21

**Authors:** Yiling Ge, Yihan Zhang, Sheng Yang, Tong Liu, Tianyi Zhang, Yanlu Feng, Chuntao Wang, Geyu Liang

**Affiliations:** ^1^Key Laboratory of Environmental Medicine Engineering, Ministry of Education, School of Public Health, Southeast University, Nanjing, China; ^2^Science and Technology Department, Jiangsu Vocational College of Medicine, Yancheng, Jiangsu, China

## Abstract

Methyltransferase-like 3 (METTL3) and methyltransferase-like 14 (METTL14) were two core components of the N6-methyadenosine (m6A) methyltransferase complex (MTC) and played a basic role in maintaining an appropriate m6A level of target genes. In gastric cancer (GC), previous researches on the expression and role of METTL3 and METTL14 were not consistent, and their specific function and mechanism have remained elusive. In this study, the expression of METTL3 and METTL14 was evaluated based on the TCGA database, 9 paired GEO datasets, and our 33 GC patient samples, and METTL3 was highly expressed and acted as a poor prognostic factor, whereas METTL14 showed no significant difference. Moreover, GO and GSEA analyses were performed, and the results pointed out that METTL3 and METTL14 were jointly involved in multiple biological processes, while they could also take part in different oncogenic pathways independently. And BCLAF1 was predicted and identified as a novel shared target of METTL3 and METTL14 in GC. In total, we conducted a comprehensive analysis of METTL3 and METTL14 in GC including their expression, function, and role, which could provide a novel insight into the research of m6A modification in GC.

## 1. Introduction

As a novel kind of posttranscriptional regulation, N6-methyadenosine (m6A) RNA modification was one of the most abundant and prevalent RNA modifications in eukaryotes [1–3]. It was a reversible and dynamic process that was installed by m6A methyltransferases “writers” and deleted by m6A demethylases “erasers” [4, 5], and m6A sites on target RNAs that recognized by m6A-binding proteins “readers” affect multiple aspects of RNA metabolism, including their transcript, splicing, processing, translation, and decay [6].

To date, a large number of related studies have focused on the relationships between m6A modification and diseases, especially cancers [7]. Emerging evidence suggested that m6A modification played a critical role in multiple cancer processes through various mechanisms [8], and alteration of m6A levels on tumor-related genes dramatically affected the cancer development, proliferation, and metastasis, [7, 9, 10]. It was reported that methyltransferase-like 3 (METTL3)-mediated m6A modification of HDGF mRNA promotes gastric cancer (GC) progression and has prognostic significance [11]. Wang et al. [12] demonstrated that YTHDF1 promoted ARHGEF2 translation and RhoA signaling in colorectal cancer (CRC), and Pu et al. [13] reported that IGF2BP2 promoted liver cancer growth through an m6A-FEN1-dependent mechanism. Moreover, m6A modification was also involved in lung cancer [14], gliomas [15], and other different cancers.

**Figure 1:**
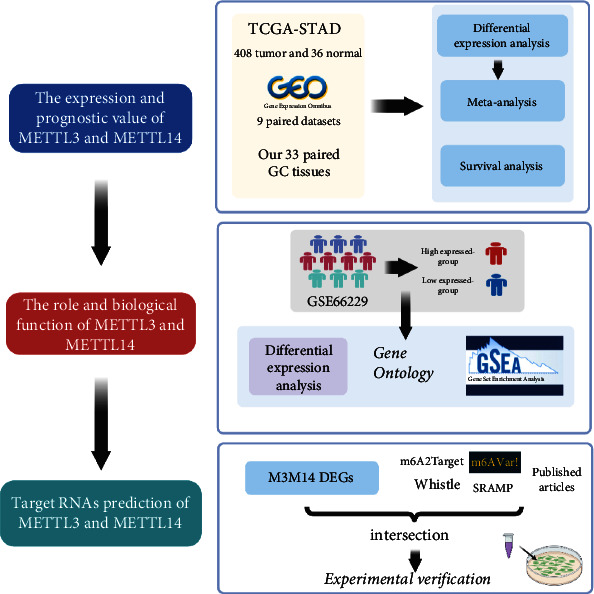
The flowchart of this study.

In the digestive system, GC was one of the most common cancers worldwide, with a high incidence rate and mortality [16]. The occurrence and metastasis of GC were considered a multifactor, multistep process, in which aberrant regulation of m6A modification was widely involved [11, 17–19]. METTL3 and METTL14 were two core components of the m6A methyltransferase complex (MTC) which was primarily responsible for m6A methylation [18, 20]. Results of crystal structure indicated that METTL3 acted as the catalytic subunit bound to the methyl donor S-adenosylmethionine (SAM) and catalyzed methyl group transfer, and METTL14 was a cofactor that was necessary for substrate RNA binding and METTL3 conformation stabilization [20–22]. In GC, one major issue concerned the existing body of research suggested that METTL3 was overexpressed and promoted GC progression [11, 19, 23, 24], while downregulated METTL14 acted as a tumor suppressor [25, 26]. These two core components of MTC paradoxically exhibited opposite expressions and roles in GC progression. Previous studies have reported dysregulation of m6A modification in GC tumorigenesis [18–20], but few types of research focused on the expression and role of METTL3 and methyltransferase-like 14 (METTL14) and their relation in the process of m6A methylation. Therefore, to elucidate the molecular mechanism of m6A modification in GC, we performed a comprehensive analysis of METTL3 and METTL14 in GC, including their expression, function, and role, as shown in Figure 1.

## 2. Materials and Methods

### 2.1. TCGA Database Analysis

GEPIA2 [27], an enhanced web (https://gepia2.cancer-pku.cn/) server for large-scale expression profiling and interactive analysis, was used to explore the expression of METTL3 and METTL14 between GC samples and normal samples in the TCGA database, and 444 eligible samples (408 GC tumor samples and 36 normal samples) were obtained. Differential expression analysis was compared by the limma package in R software.

### 2.2. GEO Database Download

Datasets in the Gene Expression Omnibus (GEO, https://www.ncbi.nlm.nih.gov/geo/) were used to explore the expression of METTL3 and METTL14. Then, “Gastric Cancer or Stomach” was selected as the keyword, and “Series (Entry type), Expression profiling by the array (Study type), *Homo sapiens* (Organism)” was used to filter the results. The datasets of GC were excluded if not meet the following criteria: (1) detection of METTL3 or METTL14 expression level in samples; (2) the dataset provided paired samples; (3) the included GC samples were all carcinoma in situ; (4) sample sizes of the dataset greater than 50. Finally, 9 datasets were obtained with the accession number: GSE29272 [28], GSE66229 [29], GSE27342 [30], GSE3438 [31], GSE29998 [32], GSE63089 [33], GSE65801 [34], GSE13911 [35], and GSE33429 [36]. Log transformation has been applied to the data of GSE27342, GSE29998, and GSE13911 samples for their high dispersion.

### 2.3. Prognostic Prediction

Kaplan-Meier (*K*-*M*) (https://kmplot.com/analysis/), one of the most comprehensive and authoritative online survival analysis websites, was used to predict the prognostic value of genes. To reveal the correlation between the expression of genes and the overall survival (OS) time of patients, the significance was computed using the Cox–Mantel (log-rank) test. The difference between the cohorts is numerically characterized by the hazard rate (HR), which is based on the differential descent rate of the two cohorts, and more information could be found by the authors of [37].

### 2.4. Gene Set Enrichment Analysis

The clusterProfiler package in R software was used to perform gene set enrichment analysis (GSEA), and h.all.v6.2.symbols.gmt was chosen as the annotated gene set. The enrichplot package in R software was used to draw the plots.

### 2.5. GO Functional Annotation

Gene ontology (GO) functional annotation was used to explore the biological function of METTL3 and METTL14. The clusterProfiler package in R software was used to perform enrichment analysis, and the result was divided into three parts, including biological processes (BP), cellular components (CC), and molecular function (MF). The enrichplot package, colorspace package, stringi package, and ggplot2 package in R software were also used. The bar plots were drawn to visualize the top results.

### 2.6. Tissue Samples from GC Patients

33 pairs of gastric and paracancer tissues were collected from Jiangsu Cancer Hospital (Nanjing, China), stored in RNA later (QIAGEN, Germany), and frozen in the freezer at −80°. All tissues were from patients who had been diagnosed pathologically as new cases of primary gastric cancer without radiotherapy.

### 2.7. Cell Lines Culture and Transfection

Human gastric mucosal epithelial cells (GES-1) and two cancer cells (AGS, HGC-27) were obtained from the Key Laboratory of Environmental Medicine Engineering, Southeast University (Nanjing, China) and certified by DNA fingerprinting. The cells were cultured in a 5% CO_2_ humidified atmosphere at 37°C. GES-1 and HGC-27 were cultured in RPMI 1640 medium (Gibco, Gaithersburg, USA) supplemented with 10% fetal bovine serum (FBS). AGS was cultured in DMEM (Gibco) supplemented with 10% FBS. The human METTL3 knockdown lentivirus, METTL14 overexpressing lentivirus, and the corresponding negative control lentivirus were synthesized by Hanheng Biotechnology Co (Nanjing, China), and the viral vector was pHBLV-CMV-MCS-3flag-EF1-ZsGreen-T2A-Puro. After 24 h of lentivirus treatment, the medium was removed and replaced with a fresh complete medium. After 72 h of lentivirus treatment, fluorescence was observed under the microscope, and puromycin was used to screen stably transfected cells.

### 2.8. RNA Isolation and RT-qPCR

Gene expression levels in tissues and cells were measured by real-time fluorescent quantitative PCR quantitative reverse transcription PCR (RT qPCR). TRIzol reagent (Invitrogen, USA) was used to extract total RNA from cells and tissues, and purity and concentration were determined using a NanoDrop 2000 spectrophotometer (Thermo Fisher Scientific, USA). RT reactions and RT-qPCR were performed using the reverse transcription system kit (GenStar, Beijing, China), and the reverse transcription procedure was accomplished in two steps. General Biotech Co., Ltd. (Shanghai, China) provided all of the RNA primers. Supplementary Table S1 listed the mRNA primer sequences for candidate genes and housekeeping genes. The comparative Ct (cycle threshold) was used for the comparison of gene expression, and the relative mRNA expression value was calculated as 40^−ΔCt^ [38].

### 2.9. Western Blotting

Western blotting (WB) was used to detect the protein level expression, cells from transfected and negative control groups were collected, and proteins were extracted using a lysate mixture (198 *μ*l RIPA and 2 *μ*l PMSF). After measuring the concentration of protein samples, they were mixed with SDS loading buffer in equal volume and adjusted to the same concentration. Acrylamide gels were prepared (TGX™ FastCast™ Acrylamide Kit), 15 *μ*l of the loading buffer was added to each well, and 15 *μ*l Maker was added to the last well for indication. 90-volt electrophoresis was performed for about 2 h, and the membrane was transferred at 70-volt for 4 h and then closed with 5% skimmed milk powder. The primary antibodies used here were shown as follows: BCLAF1(Proteintech, Cat No. 26809-1-AP), GAPDH (ABclonal, AC001), METTL3 (ABclonal, A8370), and METTL14 (ABclonal, A8530).

### 2.10. m6A Target Gene Prediction

m6A-related prediction websites were used in this study, including Whistle [39] (https://180.208.58.19/whistle/index.html), m6A Target2 [40] (m6A2Target (canceromics.org)), m6Avar [41] (m6avar-database of functional variants involved in m6A modification (renlab.org)), and SRAMP [42] (https://www.cuilab.cn/sramp/). The genes predicted in these websites were considered to be the potential targets of METTL3 and METTL14.

### 2.11. Statistical Analysis

For statistical analysis, SPSS 26.0 software (IBM Corp, USA), R software, and Excel software were utilized. All data were presented as mean ± standard deviation (SD) or median, and GraphPad Prism 9 was used for statistical analysis and graphing. Meta-analysis and forest plots were completed by Revman (Cochrane). A paired *t*-test was used to examine the differences in gene expression levels between tumor and paracancer tissues. In differential expression analysis, |log_2_ FC| > 0.05 and *P* − value < 0.01 were considered to be statistically significant. In prognostic prediction, log-rank *P* < 0.05 was considered to be statistically significant. In GO functional annotation and GSEA, adjust *P* (*P* adj) value <0.05 and false discovery rate (FDR) *q* value <0.25 were considered to be statistically significant.

## 3. Results

### 3.1. The Expression and Prognostic Value of METTL3 and METTL14 in GC

#### 3.1.1. METTL3 Was High Expressed While METTL14 Was Shown No Significant Difference

**Figure 2:**
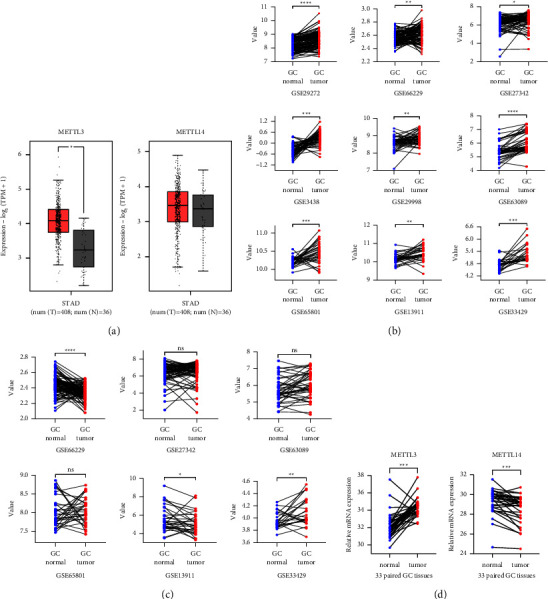
The expression exploration of METTL3 and METTL14 in GC. (a) The expression of METTL3 and METTL14 in GC from TCGA. *T*: tumor tissue; *N*: normal tissue. (b) The expression of METTL3 in GC from GEO datasets. (c) The expression of METTL14 in GC from GEO datasets. The selected samples of each dataset were paired, and all samples that lack expression were excluded. (d) The RT-qPCR results of METTL3 and METTL14 from 33 paired tissues. Value: a value that measured the relative abundance of gene transcript expression in GEO datasets.

The majority of studies reported [11, 19, 23] that METTL3 was overexpressed in GC although METTL14 was low-expressed [43]. To begin, we explored their expression in GC based on the TCGA database, which contained 408 GC samples and 36 normal samples. As shown in Figure 2(a), the expression of METTL3 was significantly upregulated (*P* < 0.01) while METTL14 showed no significant difference.

Considering the few number of normal samples from the TCGA and comparability between samples, the paired GC samples among GSE29272 [28], GSE66229 [29], GSE27342 [30], GSE3438 [31], GSE29998 [32], GSE63089 [33], GSE65801 [34], GSE1391 [35], and GSE33429 [36] were obtained from GEO to examine the expression of METTL3 and METTL14. For METTL3, all GEO datasets showed a significant upregulation in GC (Figure 2(b), Table 1). For METTL14, GSE66229 [29] and GSE13911 [35] showed a significant downregulation while GSE33429 [36] showed a significant upregulation and other datasets showed no significant difference (Figure 2(c), Table 2). In addition to public databases, 33 paired GC tissues from Jiangsu Cancer Hospital (Nanjing, China) were collected to further confirm the expression of METTL3 and METTL14, all tissues were from patients who had been diagnosed pathologically as new cases of primary GC without radiotherapy, and RT-qPCR results of our tissues showed that METTL3 was significantly overexpressed, and METTL14 was significantly decreased (Figure 2(d)).

**Figure 3:**
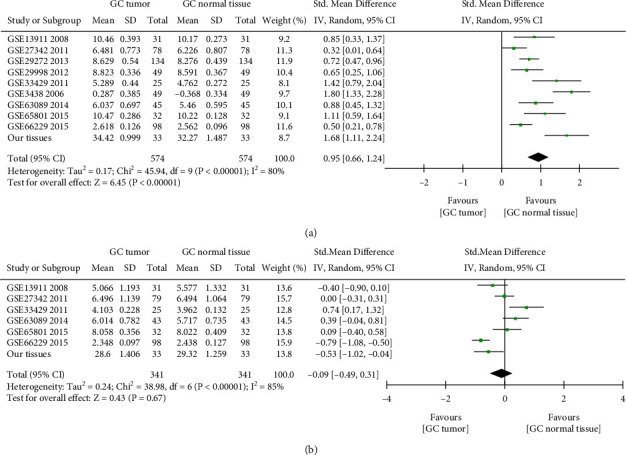
The expression validation of METTL3 and METTL14 in GC. (a) Forest plot of METTL3 expression data from GEO datasets and RT-qPCR, the pooled SMD of METTL3 was 0.95 by the random-effects model. (b) Forest plot of METTL14 expression data from GEO datasets and RT-qPCR, the pooled SMD of METTL14 was −0.09 by the random-effects model.

For the inconsistent results between datasets and our tissues, a comprehensive meta-analysis was conducted to determine the expression of METTL3 and METTL14 in GC. All GEO datasets are mentioned previously, our tissues were involved, and *I*_2_ > 50% indicated significant heterogeneity, and the random-effects model was used. The pooled standard mean difference (SMD) of METTL3 was 0.95 (95% CI: 0.66 to 1.24, *P* < 0.001) (Figure 3(a)). The pooled SMD of METTL14 was −0.09 (95% CI: −0.49 to 0.31, *P*=0.67) (Figure 3(b)). Therefore, we concluded that METTL3 was significantly overexpressed in GC, and METTL14 was low expressed but had no statistical significance.

#### 3.1.2. Prediction of Prognostic Value

**Figure 4:**
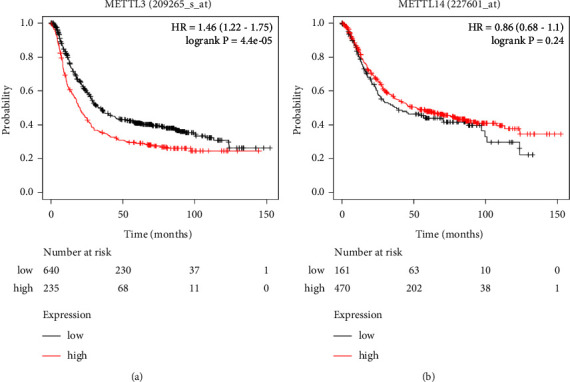
The prognostic value of METTL3 and METTL14 in GC. (a) Prognostic prediction of METTL3 in GC. (b) Prognostic prediction of METTL14 in GC. HR > 1 was shown to be negatively connected with prognosis, while HR < 1 was found to be positively correlated with prognosis.

The online bioinformatics tool K-M plotter was used to calculate the hazard ratio (HR) and *P* value for OS. 1065 patients with accompanying clinical data were included in the K-M plotter database GC cohort [44]. As shown in Figure 4, high METTL3 expression was found to be a negative prognostic factor in GC patients, with those who had higher METTL3 expression having a shorter OS time than those who had lower METTL3 expression (HR = 1.46, log-rank *P* < 0.01). Contrarily, GC patients with higher METTL14 expression had a longer OS time (HR = 0.86), but the difference showed no significance.

### 3.2. The Biological Function of METTL3 and METTL14

#### 3.2.1. M3DEGs and M14DEGs Jointly Enriched in Multiple Biological Processes in GC

**Figure 5:**
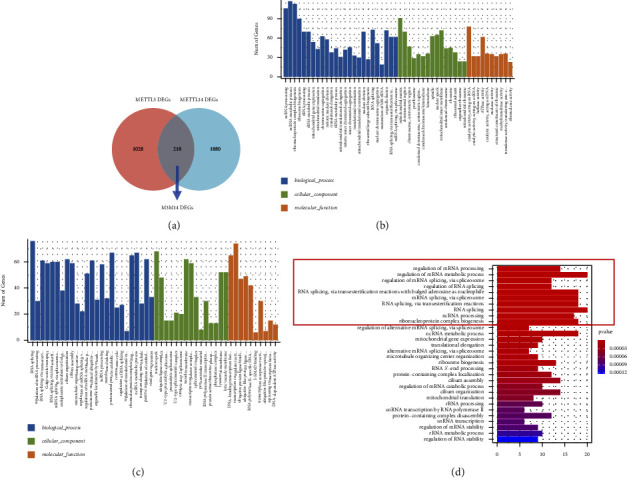
GO function annotation on M3DEGs, M14DEGs, and M3M14DEGs. (a) Venn diagrams illustrating the overlaps between M3DEGs and M14DEGs; (b)–(d) GO function annotation of M3DEGs, M14DEGs, and M3M14DEGs.

To discover more about the biological function of METTL3 and METTL14, GC patient samples in GSE66229 were chosen. The median expression of METTL3 was used to divide GC patient samples into two groups, and METTL14 handled the same. Then, differential gene analysis was performed. There were 1246 differentially expressed genes between the high- and low-METTL3 groups (METTL3 DEGs, M3DEGs) and 2098 differentially expressed genes between the high- and low-METTL14 groups (METTL14 DEGs, M14DEGs). 218 genes between M3DEGs and M14DEGs were overlapped and named M3M14DEGs (Figure 5(a)).

GO function annotation was first performed on these M3DEGs and METTL14 DEGs. The results were divided into three parts: MF, BP, and CC, and the bar plots were drawn to visualize the top results of each part (Figures 5(b) and 5(c)). As expected, the results of M3DEGs and M14DEGs were both significantly enriched in the modification and regulation of RNA, and 32 GO terms were overlapped, including regulation of mRNA processing (GO: 0050684), ncRNA processing (GO: 0034470), RNA splicing (GO: 0008380), regulation of mRNA metabolic process (GO: 1903311), ribonucleoprotein complex biogenesis (GO: 0022613), and ncRNA metabolic process (GO: 0034660). Moreover, GO function annotation of M3M14DEGs demonstrated similar results (Figure 5(d)). These results indicated that METTL3 and METTL14 jointly participated in multiple biological processes in GC, which supported the cooperative role of METTL3 and METTL14 in m6A modification.

#### 3.2.2. METTL3 and METTL14 Participated in the Specific Carcinogenic Pathways in GC

**Figure 6:**
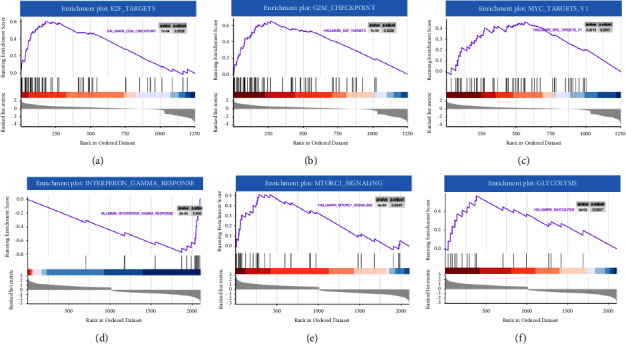
Gene set enrichment analysis of METTL3 and METTL14. (a)–(c) GSEA results of METTL3; (d)–(f) GSEA results of METTL14. NES: normalized enrichment score.

GSEA on M3DEGs and M14DEGs was also performed to gain a deeper insight into how METTL3 and METTL14 play a role in the GC process. The Hallmark gene set in the MSigDB collections was chosen as the reference gene set. As the enrichment plots shown, E2F_TARGETS (NES = 2.54, *P* adj = 0.0038, Figure 6(a)), G2M_CHECKPOINT (NES = 2.31, *P* adj = 0.0038, Figure 6(b)), and MYC _TARGETS_V1 (NES = 1.76, *P* adj = 0.0047, Figure 6(c)) were most commonly enriched in M3DGEs, showing the association between M3DGEs and these carcinogenic signaling pathways; M14DEGs was significantly enriched in INTERFERON_GAMMA_RESPONSE (NES = −2.77, *P* adj = 0.0047, Figure 6(d)) and negatively correlated with MTORC1_SIGNALING (NES = 2.31, *P* adj = 0.0047, Figure 6(e)) and GLYCOLYSIS (NES = 2.18, *P* adj = 0.0047, Figure 6(f)). These results pointed out that METTL3 and METTL14 took part in different carcinogenic pathways in GC cancer progression, which hinted that METTL3 and METTL14 might target different genes in the GC process except in addition to coworking.

### 3.3. Target Genes Prediction of METTL3 and METTL14

#### 3.3.1. BCLAF1 as a Novel m6A Target in GC

**Figure 7:**
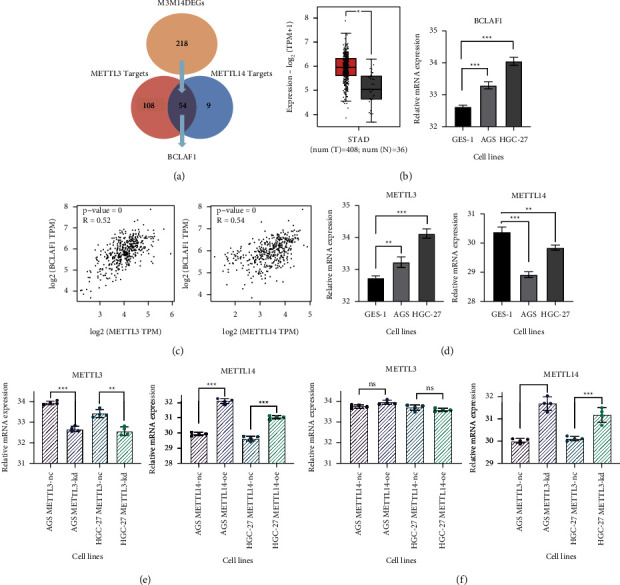
Exploration of METTL3 and METTL14 targets. (a) Venn diagrams that depicted the overlaps between METTL3 targets and METTL14 targets; (b) the expression of BCLAF1 in TCGA-STAD and GC cell lines; (c) the correlation analysis of METTL3 and BCLAF1, and METTL14 and BCLAF1 in GC; (d) the expression of METTL3, METTL14 in GC cells; (e) the validation of transfection efficiency by RT-qPCR; (f) the expression of METTL3 and METTL14 in METTL3-kd and METTL14-oe cell models.

4 m6A target prediction databases [39–42] were utilized to predict the m6A-modified genes of METTL3 and METTL14, and 54 validated targets of METTL3 and METTL14 were discovered. We intersected M3M14DEGs with 54 validated targets to get our target gene: BCL2 associated transcription factor 1(BCLAF1) (Figure 7(a)), which might be comodified by METTL3 and METTL14 in GC. BCLAF1 was significantly high expressed in GC (Figure 7(b)), and the correlation analysis between METTL3 and BCLAF1, and METTL14 and BCLAF1 in GC revealed a strong positive connection (Figure 7(c)). As a result, we hypothesized that METTL3 and METTL14 working together to produce m6A modification on BCLAF1 would enhance its expression.

**Figure 8:**
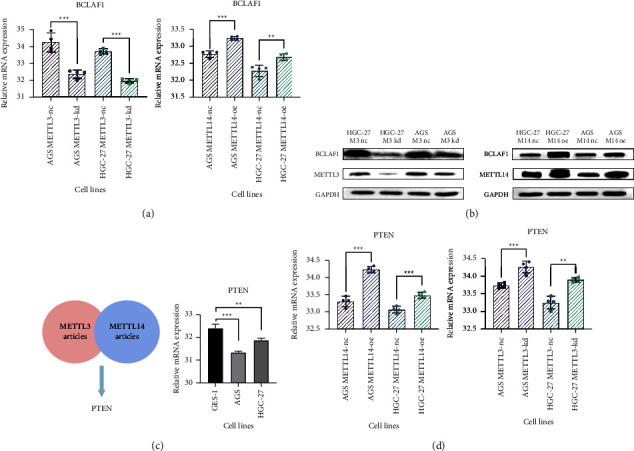
The expression validation of BCLAF1 and PTEN in transfection cell models. (a) The mRNA expression of BCLAF1in METTL3-kd and METTL14-oe cell models; (b) the protein validation of BCLAF1, METTL3, and METTL14 in METTL3-kd and METTL14-oe cell models; (c) Venn diagrams that depicted the overlaps between METTL3-related articles and METTL14-related articles; the expression of PTEN in GC cell lines; (d) the expression of PTEN in METTL3-kd and METTL14-oe cell models.

Therefore, lentiviral infection was used for the construction of cell transfection models. Based on the high-expressed METTL3 and low-expressed METTL14 in GC cells (Figure 7(d)), we established the METTL3 knockdown (METTL3-KD) cell transfection models and METTL14 overexpressed (METTL14-OE) cell transfection models in AGS and HGC-27 cell, and the transfection efficiency was verified by RT-qPCR and WB (Figures 7(e) and 8(b)). Interestingly, we found that the mRNA expression of METTL14 was upregulated after METTL3 knockdown (Figure 7(f)).

Our RT-qPCR results showed that BCLAF1 was dramatically decreased after METTL3 knockdown and was significantly upregulated after overexpressing METTL14 (Figure 8(a)). Meanwhile, these results were verified in protein level in HGC-27 and AGS cells by WB, as shown in Figure 8(b).

#### 3.3.2. m6A Modification on PTEN Mediated by METTL3/METTL14 Played an Opposite Role in Its Expression

Moreover, this study retrieved that the articles related to METTL3 or METT14 in GC, METTL3-modified genes, and METTL14-modified genes mentioned in GC articles were met in the middle (Figure 8(c), Supplementary Table S2), and phosphatase and tension homolog (PTEN) was overlapped [26, 45].

**Figure 9:**
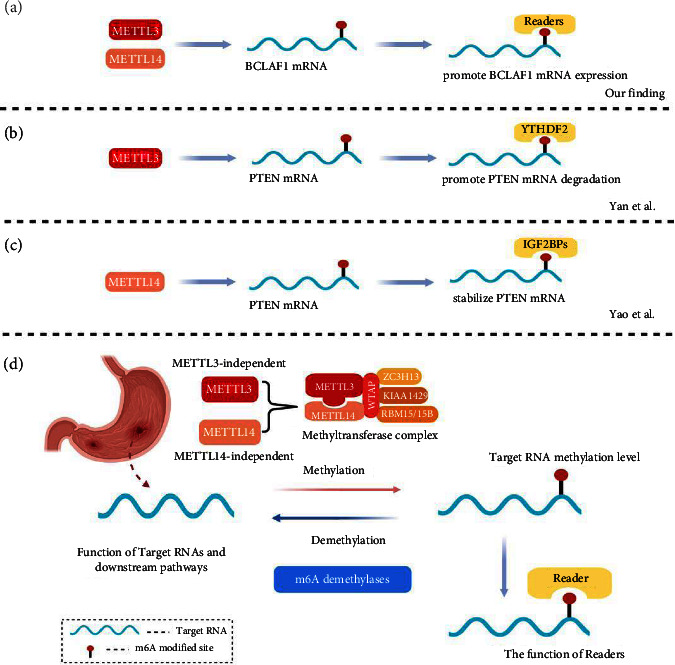
The biological function of m6A modification in the cancer progress is a multistage process and is determined by multiple factors [26, 45].

Acting as a classical tumor suppressor in the cancer process, PTEN was a key negative regulator in the PI3K signaling pathway [40]. In GC, PTEN was low expressed, and Yan et al. [45] found that METTL3 facilitated m6A-YTHDF2-dependent PTEN mRNA degradation (Figure 9(b)). Interestingly, Yao et al. [26] discovered that METTL14-mediated m6A modification on PTEN enhanced its mRNA stability (Figure 9(c)). Intrigued by these inconsistent findings, our transient transfection cell models were used to validate their findings. Knockdown of METTL3 reduced m6A modification on PTEN and significantly increased its mRNA expression, whereas overexpressed METTL14-enhanced m6A modification on PTEN also significantly increased its mRNA expression (Figure 8(d)). The fact that METTL3-mediated m6A modification on PTEN and METTL14-mediated m6A modification on PTEN played opposite effects in GC remained obscure.

## 4. Discussion

In this study, we first determined the expression of METTL3 and METTL14 in GC. A comprehensive meta‐analysis based on 9 paired GEO datasets and 33 GC tissue samples validated that METLL3 was significantly high expressed while the expression of METTL14 showed no significant difference in GC, which was consistent with the result of the TCGA database. Survival analysis results showed that METTL3 was a poor prognostic factor for GC patients while METTL14 had less prognostic value.

GO function annotation was also performed, and the result showed that M3DEGs and M14DEGs both enriched in 32 GO terms, including regulation of mRNA processing, RNA splicing, ncRNA processing, ribonucleoprotein complex biogenesis, ncRNA metabolic process, regulation of mRNA metabolic process, RNA metabolic process. It was considered that METTL3 and METTL14 jointly participated in multiple biological processes, implying the cooperative role of METTL3 and METTL14 in m6A modification.

Target genes of METTL3 and METTL14 were predicted in this study, and BCLAF1 was found. It was upregulated and reported as an oncogene [46–48]. m6A site was detected on the coding sequence (CDS) of BCLAF1 mRNA that METTL3 and METTL14 could combine with [49], and BCLAF1 revealed a strong positive correlation with METTL3 and METTL14. In our cell transfection models, the mRNA expression and the protein level of BCLAF1 were decreased after METTL3 knockdown and were upregulated after overexpressing METTL14. Taken together, we indicated that METTL3 and METTL14 jointly mediated m6A modification on BCLAF1 and promoted its mRNA expression (Figure 9(a)).

On the other hand, GSEA results demonstrated that M3DEGs and M14DEGs took part in different oncogenic pathways in the GC process, hinting that METTL3 or METTL14 might mediate m6A methylation on different target RNAs independently. PTEN was one of the classical tumor suppressors in the GC process, and the m6A modification on PTEN was deeply discussed. It was reported that METTL3 facilitated m6A-YTHDF2-dependent PTEN mRNA degradation while METTL14-mediated m6A modification on PTEN enhanced its mRNA stability (Figures 9(b) and 9(c)) [26, 45].

It was necessary to emphasize that the role of m6A modification in all cancers had two sides. According to our understanding, the biological function of m6A modification on target RNA and cancer progress was a multistage process and is determined by multiple factors (Figure 9(d)): firstly, m6A writers and erasers determined the m6A level of target RNA. Besides, the m6A level of target RNA and the m6A-binding proteins “readers” combined on target RNA determined its expression level. Finally, the own function and downstream pathway target RNA also should be considered.

Combining the GSEA results previously, we hypothesized that except for the synergistic effect of METTL3 and METTL14 on m6A MTC, each of them could mediate m6A nidification on target RNAs independently, and the m6A site could be identified by different readers under certain conditions. Therefore, the situation that METTL3-mediated RNA modification and METTL14-mediated RNA modification played an opposite role in the expression of PTEN and cancer progress could be explained.

While METTL3 and METTL14 both contained a methyltransferase catalytic domain, current theories mainly considered [20, 21] that METTL3 methylated m6A sites and METTL14 stabilized METTL3 conformation, producing m6A sites on target RNAs together. Our hypothesis needed further experimental validation and proof of chemical structure.

## 5. Conclusion

In conclusion, the present study conducted a comprehensive analysis of METTL3 and METTL14 in GC, including their expression, function, and role in GC. BCLAF1 was identified as a shared target of METTL3 and METTL14. This study provided a deeper insight into the function of m6A modification in the cancer process and hoped it could be beneficial to the mechanism exploration of m6A modification.

## Figures and Tables

**Table 1:** The expression of METTL3 from the GEO datasets.

Datasets	Country	Platform	Sample	N	Expression	*P*
GSE29272 [28] (2013)	USA	GPL96	GC tumor	134	8.629 ± 0.540	<0.001^*∗∗*^
			GC normal tissue	134	8.276 ± 0.439	

GSE66229 [29] (2015)	USA	GPL570	GC tumor	98	2.618 ± 0.126	0.001^*∗*^
			GC normal tissue	98	2.562 ± 0.096	

GSE27342 [30] (2011)	USA	GPL5175	GC tumor	78	6.481 ± 0.773	0.016^*∗*^
			GC normal tissue	78	6.226 ± 0.807	

GSE3438 [31] (2006)	South Korea	GPL2912	GC tumor	49	0.287 ± 0.385	<0.001^*∗∗*^
			GC normal tissue	49	−0.368 ± 0.334	

GSE29998 [32] (2012)	Singapore	GPL6947	GC tumor	49	8.823 ± 0.336	0.002^*∗*^
			GC normal tissue	49	8.591 ± 0.367	
GSE63089 [33] (2014)	China	GPL5175	GC tumor	45	6.037 ± 0.697	<0.001^*∗∗*^
			GC normal tissue	45	5.460 ± 0.595	
GSE65801 [34] (2015)	China	GPL14550	GC tumor	32	10.47 ± 0.286	<0.001^*∗∗*^
			GC normal tissue	32	10.22 ± 0.128	
GSE13911 [35] (2008)	Italy	GPL570	GC tumor	31	10.46 ± 0.393	0.002^*∗*^
			GC normal tissue	31	10.17 ± 0.273	
GSE33429 [36] (2011)	China	GPL5175	GC tumor	25	5.289 ± 0.440	<0.001^*∗∗*^
			GC normal tissue	25	4.762 ± 0.272	

^*∗*^ *P* < 0.01; ^*∗∗*^*P* < 0.001.

**Table 2:** The expression of METTL14 from the GEO datasets.

Datasets	Country	Platform	Sample	Number	Expression	*P*
GSE66229 [29] (2015)	USA	GPL570	GC tumor	98	2.348 ± 0.097	<0.001^*∗∗*^
			GC normal tissue	98	2.438 ± 0.127	

GSE27342 [30] (2011)	USA	GPL5175	GC tumor	79	6.496 ± 1.139	0.989
			GC normal tissue	79	6.494 ± 1.064	

GSE63089 [33] (2014)	China	GPL5175	GC tumor	43	6.014 ± 0.782	0.073
			GC normal tissue	43	5.717 ± 0.735	

GSE65801 [34] (2015)	China	GPL14550	GC tumor	32	8.058 ± 0.356	0.717
			GC normal tissue	32	8.022 ± 0.409	

GSE13911 [35] (2008)	Italy	GPL570	GC tumor	31	5.066 ± 1.193	0.041^*∗*^
			GC normal tissue	31	5.577 ± 1.332	

GSE33429 [36] (2011)	China	GPL5175	GC tumor	25	4.103 ± 0.228	<0.007^*∗*^
			GC normal tissue	25	3.962 ± 0.132	

^*∗*^ *P* < 0.01; ^*∗∗*^*P* < 0.001.

## Data Availability

The data that support the findings of this study are available from the corresponding author upon reasonable request.

## Abbreviations

*P*adj: Adjusted *P*
BCLAF1: BCL2-associated transcription factor 1
CRC: Colorectal cancer
FDR: False discovery rate
GC: Gastric cancer
GEO: Gene expression omnibus
GO: Gene ontology
GSEA: Gene set enrichment analysis
HR: Hazard ratio (HR)
mRNAs: message RNAs
MTC: Methyltransferase complex
METTL14: Methyltransferase-like 14
METTL3: Methyltransferase-like 3
M14DEGs: METTL14 differentially expressed genes
M3DEGs: METTL3 differentially expressed genes
m6A: N6-methyadenosine
NC: Negative control
ncRNAs: Noncoding RNAs
NES: Normalized enrichment score
OE: Overexpressed
PTEN: Phosphatase and tensin homolog
RT-qPCR: Real-time quantitative PCR
SD: Standard deviation
SMD: Standard mean difference
TCGA: The cancer genome atlas.

## Supplementary Materials

Supplementary Materials Supplementary Table S1. The primer sequences included in the study. Supplementary Table S2. The target gene of METTL3 and METTL14 in GC articles.
