# Supplementary material for: The Comprehensive Analysis of N6-Methyadenosine Writer METTL3 and METTL14 in Gastric Cancer
Source: J Oncol. 2023 Feb 21;2023:9822995. doi: 10.1155/2023/9822995 (PMC9974280; doi:10.1155/2023/9822995)
Supplement: Supplementary Materials — Supplementary Table S1. The primer sequences included in the study. Supplementary Table S2. The target gene of METTL3 and METTL14 in GC articles. [file 9822995.f1.docx]

**Supplementary Table S1. The primer sequences included in the study**

| RNAs | Primer sequence (5′ to 3′) |
| --- | --- |
| METTL3-F | CAAGCTGCACTTCAGACGAA |
| METTL3-R | GCTTGGCGTGTGGTCTTT |
| METTL14-F | GAACACAGAGCTTAAATCCCCA |
| METTL14-R | TGTCAGCTAAACCTACATCCCTG |
| BCLAF1-F | TCTGGAATAGAAGGCACTCTAGG |
| BCLAF1-R | ACCCTCGTCTTTTAGAAACAGGA |
| PTEN-F | TGGATTCGACTTAGACTTGACCT |
| PTEN-R | TGGATTCGACTTAGACTTGACCT |
| GAPDH-F | GGACCTGACCTGCCGTCTAG |
| GAPDH-R | GTAGCCCAGGATGCCCTTGA |

**Supplementary Table S2. The target gene of METTL3 and METTL14 in GC articles.**

| **METTL3 modified genes** | MYC, SEC62, HDGF, ZMYM1, BATF2, ARHGAP5, SEC62, CDCP1, SPHK2, PTEN, YAP1 |
| --- | --- |
| **METTL14 modified genes** | PTEN |
